# Simulated psychosis care role-plays for pharmacy curricula: a qualitative exploration of student experiences

**DOI:** 10.1007/s00127-023-02598-7

**Published:** 2023-12-16

**Authors:** Tina X. Ung, Sarira El-Den, Rebekah J. Moles, Claire L. O’Reilly

**Affiliations:** The University of Sydney School of Pharmacy, Sydney, NSW Australia

**Keywords:** Mental Health First Aid, Psychosis, Simulation, Pharmacy education, Student experience, Lived experience

## Abstract

**Purpose:**

Mental Health First Aid (MHFA) training is embedded in various tertiary healthcare curricula. However, opportunities for students to practise their newly acquired MHFA skills before entering the clinical practice workforce are lacking. The purpose of this study was to explore pharmacy students’ experiences of MHFA training and post-MHFA simulated psychosis care role-plays.

**Methods:**

Final-year pharmacy students received MHFA training, after which they were invited to participate in simulated patient role-plays with trained actors, whilst being observed by peers, pharmacy tutors and mental health consumer educators (MHCEs). Immediately after each role-play, the role-playing student engaged in self-assessment, followed by performance feedback and debrief discussions with the tutor, MHCE and observing peers. All MHFA-trained students were invited to participate in audio-recorded focus groups to explore their experiences. Audio-recordings were transcribed verbatim and thematically analysed.

**Results:**

MHFA training was delivered to 209 students, of which 86 participated in a simulated patient role-play as a role-player and the remaining students observed. Seven focus groups were conducted with 36 students (mean duration 40 min, SD 11 min). Five themes emerged: scenario reactions, realistic but not real, mental health confidence, MHFA skills application, feedback and self-reflection.

**Conclusion:**

Students enjoyed the post-MHFA simulated psychosis care role-plays, which provided opportunities to apply and reflect on their newly-acquired MHFA skills in a safe learning environment. These experiences enhanced students’ confidence to support people in the community, experiencing mental health symptoms or crises, and could be an add-on to MHFA training in the future.

**Supplementary Information:**

The online version contains supplementary material available at 10.1007/s00127-023-02598-7.

## Introduction

Pharmacists are accessible and trusted frontline healthcare professionals, with expanding roles in supporting mental health and psychological wellbeing in primary care [1–5]. Pharmacists regularly encounter people experiencing mental health symptoms or crises in the community [4, 6]. Whilst recognising their importance in supporting mental healthcare, many pharmacists do not feel adequately equipped to fulfil this role [4, 7], feeling less comfortable counselling consumers about mental health medicines compared to cardiac medicines [8]. There is a pressing need for additional mental health training in pharmacy education [8–10], and continuing professional development of pharmacists [5], to help improve pharmacists’ comfort and confidence engaging with and supporting mental health consumers and carers [2, 4, 7, 11, 12].

Mental Health First Aid (MHFA) training, available in over 20 countries to healthcare professionals and the general public [13], improves mental health knowledge [14, 15], confidence [6, 14, 16, 17], preparedness to perform MHFA behaviours [18] and decreases stigma [14, 15] amongst pharmacists and pharmacy students. MHFA training (typically 12 h) provides an action plan to approach, assess and assist a person who is experiencing mental health symptoms or crises, listen non-judgementally, give support and information, and encourage professional help and other support (ALGEE) [19]. A survey of the general public revealed that participants were more comfortable discussing mental illness and had stronger belief in pharmacists’ abilities to do so if the pharmacist had completed MHFA training [20]. Pharmacy students and staff endorse the importance of integrating MHFA training into curricula [9]. Hence, it is not surprising that MHFA training is embedded in various tertiary healthcare curricula, including pharmacy [21, 22]. However, few programme providers facilitate opportunities for students to practise their newly acquired MHFA skills post-training, which may impact future practice application [21].

In line with Miller’s pyramid of clinical competence [23], assessments involving simulated patient (SP) role-plays allow students to ‘show how’ they would apply their newly acquired knowledge, rather than self-reporting what they would do in a fictional scenario (i.e. ‘knows how’) [16, 17]. Through SP role-plays, learners can practise skills with real people and receive feedback on their performance [24], whilst being encouraged to simultaneously reflect on their own practice [25]. SP assessments must be holistic, authentic and realistic in order to meet societal needs and optimise outcomes for mental health consumers/carers [9]. Authentic scenarios, reflective of real-world practice, can be created by collaborating with mental health stakeholders, such as consumers and mental health professionals, for assessment delivery and evaluation [26, 27]. Most mental health simulations in pharmacy education and practice, as well as MHFA evaluations, have demonstrated greater self-reported preparedness of participants to provide MHFA to people experiencing depression or anxiety. Currently, there is less evidence evaluating SP scenarios for psychosis, substance use disorders, or eating disorders [28]. Furthermore, there is evidence pharmacists’ knowledge and literacy surrounding psychosis is lacking, with symptoms of schizophrenia less easily recognised by pharmacists than depression [29]. Therefore, the aim of this study was to qualitatively explore pharmacy student experiences of MHFA training and post-MHFA simulated psychosis care role-plays.

## Methods

This qualitative study applied an inductive thematic analysis approach to analyse student feedback on MHFA training and simulated psychosis care role-plays, collected via focus group discussions.

### Study intervention

Twelve hours of MHFA training was delivered to all final-year Bachelor of Pharmacy (BPharm) and Master of Pharmacy (MPharm) students at The University of Sydney, in workshop groups of approximately 20 students each. MHFA training is embedded into the final-year timetable of the 4 year undergraduate BPharm and 2 year postgraduate MPharm degrees, completed in 1.5 days and over four 3 h sessions, respectively. Post-MHFA training, each of the same MHFA workshop groups attended a 90 min compulsory workshop, with students invited to participate in SP role-plays with trained actors. Three previously co-designed and content validated simulated psychosis care scenarios and associated marking rubrics [27, 30] (relating to a person experiencing first episode psychosis with suicidal thoughts, a carer of someone living with schizophrenia, non-adherence to antipsychotic medicine) were used. Their development in partnership with mental health consumers and professionals is described elsewhere [27]. Students in each workshop were divided into three groups, with whom each of the three scenarios was enacted by a trained actor with a role-playing student, observed by peers, a MHFA-trained tutor and a mental health consumer educator (MHCE). Following each role-play, the role-playing student engaged in self-assessment using the content valid marking rubric pertaining to that scenario, followed by performance feedback and debriefing with peers, the MHCE and tutor.

Actors engaged in a training session (30–60 min) with two members of the research team (TU, who is a MHFA-trained pharmacist, and SE, a pharmacist and Master MHFA instructor) to rehearse their allocated scenario beforehand, to ensure consistency. MHCEs were recruited via not-for-profit organisations One Door Mental Health and Bipolar Australia [31, 32].

### Study procedures and data analysis

A semi-structured focus group guide was developed, based on researchers’ previous experience and literature [33]. All students were invited, pre- and post-MHFA training via the students’ learning management system online and in-person, to participate in a focus group facilitated by TU. The focus groups were digitally recorded and transcribed verbatim by TU. Guided by Braun and Clarke’s step-by-step guide to conducting thematic analysis [34], all transcripts were de-identified, then read and re-read by TU for data familiarisation. Initial codes were generated, and preliminary themes mapped by TU using Miro software [35]. A second author (COR, a Master MHFA instructor and pharmacist) independently read and coded 25% of transcripts. Themes and subthemes were iteratively discussed and reviewed, then defined and refined collaboratively with two other co-authors (SE and RM).

### Ethics approval and reporting

This study was approved by the Human Research Ethics Committee of The University of Sydney (Project No. 2021/507) and reported in accordance with Standards for Reporting Qualitative Research [36]. All participants provided written informed consent prior to SP workshop and focus group attendance.

## Results

Of 209 MHFA-trained students, 86 participated in a SP role-play across 11 workshop sessions, whilst the remaining students observed. The role-plays and focus groups were conducted with MPharm students in May 2022 and BPharm students in October–November 2022, post-MHFA training. Due to scheduling, the focus groups were conducted either immediately post-workshop or 7–10 days post-workshop. Seven focus groups were conducted (two with MPharm, five with BPharm) with a total of 36 students (mean duration 40 min, SD 11 min). Mapping of initial codes and preliminary themes generated from the focus group transcripts is shown in Appendices (Supplementary files) 1 and 2. Five overarching themes emerged: scenario reactions, realistic but not real, mental health confidence, MHFA skills application, feedback and self-reflection.

### Scenario reactions

Students described the scenarios as *‘intense and confronting’* [FG1P5] and ‘*shocking’* [FG5P4] for both role-players and observers, who had not experienced these situations before. However, students valued the exposure in preparation for future practice:

*‘… now I've somewhat been exposed. So, like, it would still be a shock, but at least it has helped to show what it could be like… So at least I can somewhat be of help.’* [FG5P2]

Exposure to the psychosis care scenarios within the safe environment of the classroom gave students a realistic *‘taste of what could definitely happen in real life’* [FG1P2]:

*‘It’s good exposure therapy, especially for our professional expectations in the future, because there's a very real chance we're going to be interacting with these people.’* [FG1P6]

The scenarios challenged personal beliefs and stigma about people experiencing mental health symptoms and crises, especially students’ misconceptions of people living with schizophrenia:

*‘…I immediately just thought they would be, like, crazy and, like, yelling and stuff. That's where my brain immediately went. And then she was just like calm…and easy to chat to. And I was not expecting that.’* [FG5P2]

Students shared the *‘taboo’* [FG1P5] nature of mental health in their cultures; the role-plays challenged them to experience and interact with mental health scenarios for the first time:

*‘…where I come from…I haven't had connections with people who have mental health or even know that they have these issues. So putting myself out there and being able to talk to these people for…the very first time…was a great opportunity to…realise, you know, it's not a big taboo. It's just part of life.’* [FG1P5]

### Realistic but not real

Students unanimously voiced that the SP scenarios were authentic and realistic; role-playing with trained actors provided an enjoyable, immersive experience to practise MHFA skills in a safe learning environment. The scenarios were *‘situations that could occur in real life*’ [FG5P3] and ‘*representative of what actually happens’* [FG1P1], especially when initiating conversation about sensitive topics. Furthermore, students reflected that these scenarios enabled them to practise how to gather information to assess for possible medication non-adherence:

*‘…most often than not, you're going to have a patient that comes in and isn't ready to say everything to you, isn't ready to divulge everything. So you have to do a bit of digging.’* [FG1P1]

Role-playing with an unfamiliar SP actor challenged students to modify their tone and language as though communicating with a real lay person:

*‘I can have a really relaxed conversation, make it more like personable to them... this is a patient… I was like, how would I deal with this in real life?’* [FG2P1]

Whilst perhaps *‘a lot more confronting’* [FG3P4] than role-playing with peers, engaging with SP actors provided a beneficial learning experience:

*‘Just made it more realistic, better practice than talking to the person who sits next to you every class.’* [FG2P4]

Interacting with SP actors was superior to role-playing with familiar tutors, who might go in and out of character and *‘steer’* [FG4P1] students in a particular direction:

*‘With actors…they don't really have any loyalty towards you… So I thought it was more realistic and more engaging and probably a better experience to have it be done with an actor as opposed to just the tutor.’* [FG4P1]

The SP role-play experience was compared with preparing for and participating in an objective structured clinical examination (OSCE) or a marked assessment, which students described to be *‘very scripted, very fake…no emotion in it’* [FG5P3]. However, trained actors enabled genuine engagement with the scenarios:

*‘Before I started it, like the role-play…I was trying to like plan it out almost like it would be an OSCE or something that's getting marked, and like what the correct solution is and how to get to it… Because it was a real actor, I really quickly forgot that. And it was almost like I was just there talking to someone who was actually having this problem.’* [FG2P2]

Whilst students valued the authenticity of the scenarios and the opportunity to practise their MHFA skills in a safe learning environment, they were still aware that the experience was simulated and may be doubtful of their translation into real-life practice:

*‘It's difficult to say whether or not we'd actually put into effect… Here we always knew that we were safe...’* [FG4P1]

Prior knowledge and expectation of a mental health simulation may also have impacted performance behaviour versus actual practice behaviour, especially when negotiating privacy for conversations relating to mental health:

*‘We still have that knowledge that it's not real, real…because we knew it was Mental Health First Aid the person was like straightaway, “Do you want to go somewhere more private?”’* [FG4P5]

### Mental health confidence

The SP role-plays enhanced, for both role-playing and observing students, confidence to support people experiencing mental health symptoms or crises:

*‘Definitely increased my confidence…if I have never encountered this kind of scenario, I'll be doubting myself or I'll be feeling nervous because I don't know where to start or how to start.’* [FG2P3]

Students acknowledged that knowledge (what to say) together with application (how to say it) contributes to confidence:

*‘…with the knowledge you’re already, already halfway there, in terms of confidence, but the thing that boosts it is when you actually practise it and you feel the adrenaline and you feel the emotions when you're being confronted with scenarios like that.’* [FG5P1]

Feedback from people with lived experience was also important in improving confidence:

*‘Being put in that situation and…getting people who have actually experienced it say that, you know, it’s okay for you to ask those questions and getting that validation…made me a lot more confident…’* [FG6P3]

Despite acknowledging enhanced mental health confidence, some students still held doubts regarding the application of MHFA skills in real-world settings:

*‘…I feel confident that I have the skills and I know the things that I'm meant to say, but my biggest concern is thinking about how the person is going to react to the things that I'm saying… I always assume that they might take it the wrong way or they're a bit scared, I feel I impose myself from doing it… if I had more confidence that things don't always go wrong, and that sometimes it really does help, and they are willing to have that conversation, then I'd be more inclined to do it personally.’* [FG4P1]

Students provided suggestions on how their mental health confidence could be further improved. Given the engaging nature of the SP scenarios, students would appreciate *‘repeated exposure’* [FG4P1] and more opportunities to apply their MHFA skills:

*‘…applying the knowledge is something that I think I guess with a lot of us, is something that we're not too confident about. And I feel like this role-play actually really helped with that. And I feel like if I did more role-plays then I'd feel like I'd be more confident.’* [FG3P1]

To further improve mental health confidence, especially in preparation for clinical placements, students expressed the desire to receive MHFA training earlier in the degree:

*‘I do feel like this [MHFA] course is introduced a bit late…’ *[FG5P4]

Students also requested that more education and guidance on *‘how to use the [mental health] resources, and then how to navigate through those resources’* [FG6P4] might help to further enhance confidence to support people experiencing mental health symptoms or crises:

*‘I think it might be interesting to have some kind of mental health workshop where you've got different services telling you about what they do.’* [FG4P5]

### MHFA skills application

Students identified that the ALGEE action plan provided in MHFA training gave them a structure to remember and follow, and ultimately confidence, to approach different mental health scenarios:

*‘After the training I felt like, there's more of a framework that I can follow so it'll be easier for me to navigate that compared to like when I don't have any knowledge at all.’* [FG7P1]

Improved confidence enabled students to apply their newly-acquired MHFA skills in the SP role-plays, using the framework provided in MHFA training.

*‘…we know what it is now, so we can actually identify it and kind of go through it. And then the tutors are here and the people who have that experience to tell us step-by-step on how to navigate through that.’* [FG6P5]

The SP role-plays were more engaging and memorable than information delivered from didactic lectures or MHFA training alone. The SP role-plays ‘*solidified’* [FG2P4] MHFA information, committing both clinical and practical skills to memory:

*‘It is one thing to learn but another thing to actually implement. Do it yourself and that's how you actually stick to it.’* [FG6P6]

Students had previously *‘froze in that moment’* [FG4P1] and felt uncomfortable identifying, approaching, helping or managing mental health scenarios (such as whilst on clinical placement or in their current employment), especially around suicide. MHFA training and the opportunity to practise suicide assessment skills in the classroom enhanced confidence for such scenarios in the future:

*‘I'd actually like had a patient come up to me and tell me that they wanted to end their life… I didn't have that training then… How can I improve for next time, make sure you know, be more prepared rather than just be blindsided like that.’* [FG5P3]

A key message that students took away from MHFA training and SP role-plays was the importance of asking a person directly if they are having suicidal thoughts:

*‘The best thing to do is be direct. So like I think it's important to know… I wouldn't have known otherwise.’* [FG2P1]

The experience and learnings from participating and/or observing MHFA role-plays was reported to be translatable to future clinical practice:

*‘I know if this was to happen in a pharmacy setting, like, I'll know what to do.’* [FG3P5]

### Feedback and self-reflection

Immediately after each SP role-play, the role-playing student engaged in self-assessment using the purposefully developed rubric pertaining to that scenario, followed by feedback and debriefing with the MHCE, tutor and peers. Students saw the value in immediate self-assessment, recognising that they are their own harshest critic:

*‘…most of the time you step yourself down quite a lot... And then when you actually get the feedback from the consumer, you feel like, oh, I’m actually not doing that bad…so it builds confidence back…’ [FG5P4]*

The importance of self-reflection was highlighted; students recognised that reflecting on their performance via self-assessment, alongside feedback and debrief discussions, would impact future practice. If given the ‘*opportunity to do it a second time, which I probably will in my career, I would do better.’* [FG6P1]. Students acknowledged the role of reflective practice in continuing professional development and self-improvement:

*‘…in practice as well, no one's going to be able to tell you did this right, didn't do this right. So getting you to think self-reflectively first is a good thing… Once we finish, once we’re in our own practice to then self-assess because it's ongoing self-assessment, self-improving.’* [FG1P1]

The self-assessment and feedback process enabled students to triangulate feedback from the tutor, lived experience perspective from the MHCE, and rubric criteria:

*‘…link up what the individual with lived experience was saying, with what the sort of fundamental criteria [in the rubric] are and helping develop that sort of process in our minds...’* [FG4P1]

Students especially valued receiving feedback and learning from MHCEs, who provided realistic context from their personal experiences, complementing and going beyond the marking rubric criteria for a beneficial learning experience:

*‘When you get feedback from tutors it's more like “How many marks did I get? Did I pass or fail?” But then there are things that the consumer will tell you that's not in the rubric and I think that's what's really valuable.’* [FG5P1]

Students reflected on the value of feedback provided by MHCEs, which some felt more important than feedback provided by their tutor:

*‘I would probably value that feedback more than a marker, to be honest, because... These are…real people and the ones we’ll be talking to.’* [FG1P2]

Whilst receiving feedback from MHCEs was beneficial, students voiced an understanding that consumer feedback was from individual experiences that may not be generalisable to all mental health scenarios:

*‘…whilst it was useful in that particular context, it's hard to say if, you know, we sort of took it on board and applied it in the next situation that we thought was similar, it was going to be equally effective as what they were saying.’* [FG4P1]

Students might have felt reluctant to role-play, or ‘*scared to have that conversation’* [FG4P1], however they reflected that conducting the role-plays in a group with debrief discussions helped prepare them for when they encounter similar scenarios, on their own in real-life, with actual health implications for the patient:

*‘I get really scared... But I realised that I'd rather like this happened rather than me do it on my own, because I feel like we had a sort of discussion afterwards about how my performance was, which I found really helpful… they were telling me what I could have done better and what I did good at. And having that kind of debrief afterwards, I felt really helped consolidate what I kind of learnt and what I should do and seeing how other people dealt with their situations and role-play I felt was really good as well, because I also took away from them what I can apply for my own as well.’* [FG6P3]

## Discussion

This study demonstrated that psychosis care SP scenarios, developed through co-design with MHCEs and content validation with mental health stakeholders, are authentic and relevant to pharmacy practice. SP role-plays allowed students to apply their newly-acquired MHFA skills in a safe learning environment. The realism of the scenarios was enhanced by engaging trained actors to portray the SPs, fostering an enjoyable and immersive experience for the students. The scenarios were at times confronting, but together with a purposefully designed and structured rubric, as well as opportunity to learn from and debrief with MHCEs, tutors and peers, they improved students’ mental health confidence for future practice.

Much of the qualitative results concur with previous literature exploring participant opinions of MHFA training; completing MHFA is more valuable than didactic education about mental illnesses and evidence-based treatments [4, 15, 37]. Whilst didactic methods of education may equip pharmacists with knowledge and desire to provide medicines information, participants report lacking confidence and skills necessary for problem-solving and communicating effectively with mental health consumers/carers [9, 37]. Interventions that allow participants to apply desired behaviours, such as via SP role plays, are effective in further improving confidence and enable acquired skills to be integrated into clinical practice [25].

Encountering mental health crisis situations in a pharmacy can be a challenging experience for pharmacy staff; many express uncertainties about their level of training in how to initiate and conduct useful conversations that would not cause discomfort, further distress or harm amongst consumers [3, 7]. As previously demonstrated with medical students practising clinical communication with SPs [25], the unexpected content of the SP scenarios in this study challenged students, however they were reflective of real-world experiences in community pharmacy. The authenticity of the scenarios, delivered in a safe learning environment, allowed students to engage in active learning to apply their skills and self-reflect on aspects that could be improved for future interactions with real patients.

One particular aspect that students commonly voiced increased confidence in, post-MHFA training, was asking about suicidal thoughts. This result concurs with students in a previous study who completed MHFA training as part of an elective, who reported increased comfort in talking about mental health and suicide, awareness of signs and symptoms of common mental health disorders, and increased comfort in providing care for an individual with a mental health diagnosis and identifying signs in themselves [38]. When evaluating MHFA training with pharmacists, the largest effect was also in their reported confidence in asking if someone is having suicidal thoughts [6]. Post-training enhancement of skills and confidence to conduct suicide assessment has furthermore been observed in other behavioural assessment literature beyond pharmacy, such as with social work students [39], in programmes involving social work, midwifery, special needs education and nursing [40], university employees [41], and university hospital staff [42].

The SP role-plays provided a positive experience for students’ skill development, regardless of whether they participated in or observed the scenarios. Consistent with broader literature in this area, this suggests live simulation is critical to the MHFA training experience [4], as even observing other students providing MHFA and communicating with SPs about mental health symptoms and crises is a beneficial learning experience for students, which has demonstrated a sustained improvement in confidence post-MHFA training [43]. Concurring with the results of this study, medical students perceived clinical communication training with SP actors more realistic, convincing, safe, motivating and engaging, than role-playing with student peers, both practically and theoretically; role-playing with a familiar person often feels artificial [25]. Additionally, the theme of feedback and self-reflection emerging from this study concurs with previous research with pharmacy students who participated in suicide crisis scenarios. Being able to observe and engage in feedback and debrief discussions after role-plays allows active self-reflection and professional development [17, 24]. Furthermore aligning with the aforementioned studies, students in this study acknowledged the significance of a teacher’s feedback and how it enhanced future contacts with real patients [25], also appreciating the opportunity to receive feedback from someone with a lived experience of mental illness about how they could improve their MHFA and communication skills [17].

When discussing how mental health confidence could be further improved, students suggested introducing MHFA training earlier in their degree, especially since some had already encountered mental health scenarios during clinical placement or in their employment alongside pharmacy coursework. MHFA may be integrated towards the end of healthcare curricula as close as possible to graduation so that students are equipped with necessary mental health skills as they embark on future practice [22]. On the other hand, it is suggested that training university students in the first year of their degrees may equip them to provide peer-to-peer support during their time at university [22], and according to a pharmacy programme representative that offers MHFA training during the internship (fifth) year, students may have informal opportunities to practise MHFA *‘in the course and more so in their jobs’.* [21]. As voiced by participants in this and other studies evaluating MHFA training [44], pharmacy professionals need more training in navigating mental health resources. It is recommended that participants acquire more communications training, experiential learning and interprofessional education to improve mental health confidence [9].

A major strength of this study was that students experienced a range of scenarios simulating a person requiring assistance and support relating to psychosis care, which they may encounter in future clinical practice. Engaging trained actors to portray the SPs provided a realistic and immersive experience; actors rehearsed their roles with two members of the research team for consistency in delivery. As for study limitations, social desirability response may have been involved during focus groups, as they were conducted by the same researcher (TU) who was a tutor involved in the SP sessions. However, participation in this study was voluntary and responses were de-identified.

Future work evaluating MHFA training, and the application of MHFA skills via the SP methodology is warranted, both locally and internationally. Whilst MHFA training is available to the general public as well as health professionals, there remains a lack of public awareness of pharmacists having MHFA training [20]. Therefore, it is recommended that MHFA-trained pharmacists promote their credentials within their communities. MHFA accreditation lasts 3 years; there is limited evidence to demonstrate the sustainability of improvements up to 6 months, and no studies have reported findings beyond 1 year post-MHFA training [14, 22]. Longitudinal studies are needed to investigate whether MHFA-trained participants’ crisis assessment skills change over time [3], which may also be evaluated via SPs. It is also suggested that studies use a control or comparison group to test the independent impact of MHFA training and/or SP role-play assessments on intervention behaviours [18]. Additionally, it is not clear if the evidence base of perceived confidence and ability to apply MHFA at the tertiary student level is transferable to health professionals in practice [2, 45]. Based on the results of students desiring MHFA training earlier in their degree, future studies may compare the sustainability of acquired skills when these students graduate as practising pharmacy interns and/or registered pharmacists. Due to ethical considerations, it would be difficult to observe and report on behaviours in real-life mental health scenarios in clinical practice, also not possible to predict when such a scenario would present in real-life practice. Future research should involve examining if behaviours in SP role-plays translate to real-life practice, by means of involving mental health crises in real-world community pharmacy settings using the SP methodology, where trained actors present at community pharmacies and report on the experience [3, 6].

## Conclusion

This qualitative study has demonstrated that students valued participating in and/or observing simulated psychosis care scenarios post-MHFA training. Engaging trained actors to enact SPs enhanced the realism of the role-plays, and students recognised the importance of receiving performance feedback and learning from MHCEs, to support their confidence in caring for people experiencing mental health problems and crises. Such observed behavioural assessments may complement current evidence of MHFA training to further improve mental health confidence and allow skills application and self-reflection. It is recommended that future studies involve evaluating self-reported MHFA behaviours and confidence in real-world community pharmacy settings.

### Supplementary Information

Below is the link to the electronic supplementary material.

Supplementary file 1 (JPG 24642 KB)

Supplementary file 2 (DOCX 12 KB)

## Data Availability

The datasets generated during and/or analysed during the current study are not publicly available as consent was not obtained for public sharing of data.
