# Supplementary material for: Simulated psychosis care role-plays for pharmacy curricula: a qualitative exploration of student experiences
Source: Soc Psychiatry Psychiatr Epidemiol. 2023 Dec 16;59(7):1269–77. doi: 10.1007/s00127-023-02598-7 (PMC11178615; doi:10.1007/s00127-023-02598-7)
Supplement: Supplementary file 2 — (DOCX 12 KB) [file 127_2023_2598_MOESM2_ESM.docx]

**Appendix 2** Abbreviations used in mind map

env=environment; exp=experience; fam=family; fbk=feedback; intx=interact; LEE=lived-experience educator; MH=mental health; MHFA=Mental Health First Aid; OSCE=objective structured clinical examination; ppl=people; profs=professionals px=patient;

Q=question; re=regarding; RP=role-play; UoS=unit of study; vs=versus; w/=with.
